# Understanding geographic variations in psychiatric inpatient admission rates: width of the variations and associations with the supply of health and social care in France

**DOI:** 10.1186/s12888-018-1747-2

**Published:** 2018-06-05

**Authors:** Coralie Gandré, Jeanne Gervaix, Julien Thillard, Jean-Marc Macé, Jean-Luc Roelandt, Karine Chevreul

**Affiliations:** 1 ECEVE, UMRS 1123, Université Paris Diderot, Sorbonne Paris Cité, INSERM, Paris, France; 2 AP-HP, URC-Eco, DHU PePSY, Paris, France; 3 National Conservatory of Arts and Crafts, LIRSA, EA 4603 Paris, France; 4 World Health Organization Collaborating Centre for Research and Training in Mental Health, Lille, France

**Keywords:** Inpatient care, Geographic variations, Supply of care

## Abstract

**Background:**

Inpatient care accounts for the majority of mental health care costs and is not always beneficial. It can indeed have detrimental consequences if not used appropriately, and is unpopular among patients. As a consequence, its reduction is supported by international recommendations. Varying rates of psychiatric inpatient admissions therefore deserve to draw attention of researchers, clinicians and policy makers alike as such variations can challenge quality, equity and efficiency of care. In this context, our objectives were first to describe variations in psychiatric inpatient admission rates across the whole territory of mainland France, and second to identify their association with characteristics of the supply of care, which can be targeted by dedicated health policies.

**Methods:**

Our study was carried out in French psychiatric sectors’ catchment areas for the year 2012. Inpatient admission rates per 100,000 adult inhabitants were calculated using data from the national psychiatric discharge database. Their variations were described numerically and graphically. We then carried out a negative binomial regression to identify characteristics of the supply of care (public and private care, health and social care, hospital and community-based care, specialised and non-specialised care) which were associated with these variations while adjusting our analysis for other relevant factors, in particular epidemiological differences.

**Results:**

Considerable variations in inpatient admission rates were observed between psychiatric sectors’ catchment areas and were widespread on the French territory. Institutional characteristics of the hospital to which each sector was linked (private non-profit status, specialisation in psychiatry and participation to teaching activities and to emergency care) were associated with inpatient admission rates. Similarly, an increase in the availability of community-based private psychiatrists was associated with a decrease in the inpatient admission rate while an increase in the capacity of housing institutions for disabled individuals was associated with an increase in this rate.

**Conclusions:**

Our results advocate for a homogenous repartition of health and social care for mental disorders in lines with the health needs of the population served. This should apply particularly to community-based private psychiatrists, whose heterogeneity of repartition has often been underscored.

**Electronic supplementary material:**

The online version of this article (10.1186/s12888-018-1747-2) contains supplementary material, which is available to authorized users.

## Background

Mental disorders are severe and disabling disorders which are estimated to affect one out of four individuals over the course of life [1], with significant consequences in terms of quality of life [2], life expectancy [3–6] and disability [7]. They are also associated with considerable costs as spending linked to such disorders account for 3 to 4% of the gross national product of developed countries [8]. Inpatient care is responsible for the majority of those costs [9, 10] and is not always beneficial for patients. This type of care can indeed have detrimental consequences if not used appropriately, such as loss of autonomy or isolation, and is unpopular among patients [11–13]. As a consequence, its reduction is supported by international recommendations for mental health care [14–16] and would contribute to limit the economic burden associated with psychiatric disorders [17].

Given those elements, varying geographic rates of psychiatric inpatient admissions deserve to draw attention of researchers, clinicians and policy makers alike as such variations can challenge quality, equity and efficiency of care. However, few studies have focused on practice variations in psychiatry [18]. If previous research has underscored significant geographical variations in psychiatric inpatient admission rates in different contexts [19–25], such research has only been limited to a few geographic areas. Moreover, while direct characteristics of population health needs, related to their health status, and socio-economic factors were shown to be associated with psychiatric inpatient admission rates [12, 23, 25–27], research aimed at understanding the other factors associated with their variation remains scarce. This is in particular the case for the characteristics of the supply of care which have only been included by a limited number of studies that focused only on specific care providers due to the lack of availability of complete data sources [23–25, 28].

In this context, the development of additional studies aimed at describing geographic variations in psychiatric inpatient admission rates on a large scale and at understanding their associations with a wide range of characteristics of the supply of care, which can be targeted by dedicated health policies, appears essential. Our objectives were therefore first to describe geographic variations in inpatient admission rates across the whole territory of mainland France, and second to identify the characteristics of the supply of care which were associated with these variations after adjusting for other factors, and in particular epidemiological differences.

## Methods

### Setting

Our study was carried out on the whole territory of mainland France for the year 2012. It focused on public psychiatry as it employs a large part of psychiatrists [29] and accounts for nearly 70% of the costs of mental health care in France [9]. The majority of these costs are covered by the French universal social health insurance (SHI) system, which limits financial barriers to accessing public psychiatric care. However, a small proportion of inpatient care is not reimbursed by the SHI. Most of the population subscribes to private complementary voluntary health insurances to cover these cost-sharing obligations, with specific measures implemented to enable people with low incomes to receive free or low-cost supplementary private health insurance. A long-term illness scheme was also created to support patients with chronic disorders including severe mental illnesses. Patients in this scheme are exonerated from co-payments of any health care linked with their chronic illness [30–32].

To describe and analyse variations in psychiatric inpatient admission rates, we had to carry out our analysis between comparable areas. In France, public mental health care is organised separately for adults, children and adolescents, and forensic patients. For these three populations, there is a territorial organization of care through the psychiatric sector system. Such sectors are multidisciplinary teams providing integrated outpatient and inpatient care necessary to cover the mental health needs of the population of a defined geo-demographic area (sector’s catchment area) [33]. The staff needed to provide these services is employed by a hospital which can be in charge of several sectors. This hospital may be either a public hospital or a private non-profit hospital fulfilling public duties, and it can also be either specialised in psychiatry or a general hospital with an activity in psychiatry. Additionally, complementary care may be delivered in sectors’ catchment areas by social institutions or private providers, either specialised (such as community-based private psychiatrists, psychologists or private for-profit hospitals) or not (such as general practitioners).

Due to those specificities, our study was carried out in adult non-forensic sectors’ catchment areas on the whole territory of mainland France. To ensure comparability and data quality, we only included data from sectors linked to hospitals which provided exhaustive information on their inpatient admissions. These sectors were identified by cross-checking aggregated data from the French national discharge database which contains individual information on the use of psychiatric care (*Recueil d’informations médicalisé en psychiatrie*, RIM-P) [34] with data from the annual national survey on health care providers (*Statistique annuelle des établissements de santé*, SAE) [35]. Access to the RIM-P database requires an authorization from the French data protection authority (CNIL) that we obtained in August 2014 (Decision DE-2014-090).

We built sectors’ catchment areas using patient-origin data to take into account actual patients’ behaviours when seeking care (as some patients can choose to visit a different sector than the one they are supposed to be treated in) [36]. Based on the official age limit of adult psychiatry in France [37], the zip codes of patients aged over 16 seen in each psychiatric sector were extracted from the RIM-P database. A geographic information system (Geoconcept® software) was then used to map the catchment areas and to exclude outliers’ zip codes.

### Inpatient admission rate

The number of inhabitants in each sector’s catchment area admitted in inpatient care at least once in each sector over the course of the year studied (2012) was extracted from the RIM-P database. We only considered inpatient admissions in full-time care (day and night) provided in hospital settings. They indeed represent the most costly form of inpatient care both in France and abroad [9, 10, 38, 39], have the most prejudicial consequences for patients [14–16] and are the most studied and comparable across countries [12, 22–24]. We therefore excluded full-time care provided outside of inpatient settings, such as home-based hospitalizations or psychiatric rehabilitation centres, as well as part-time hospitalisations (day or night hospitals).

We considered inpatient admissions of patients who were diagnosed with a mental disorder from Chapter V of the International Classification of Diseases (ICD-10) [40]. We excluded inpatient admissions of patients suffering from organic mental disorders, mental retardation and disorders of psychological development (apart from pervasive developmental disorders). This diagnosis scope corresponds to psychiatrists’ expertise in France and has been used in previous international studies in the mental health field [24, 41, 42].

The inpatient admission rate was calculated per 100,000 inhabitants of sectors’ catchment areas by dividing the number of inhabitants admitted in inpatient care at least once over the course of the year 2012 in each sector by the total number of inhabitants aged over 16 in the sector’s catchment area. Patients with multiple inpatient admissions were therefore only counted once.

### Potential factors associated with geographic variations in inpatient admission rates

#### Epidemiological factors

To account for epidemiological differences in sectors’ catchment areas which can be associated with warranted variations in inpatient admission rates, we first considered several direct characteristics of population health needs. They included two characteristics of the mental health status of the population living in each sector’s catchment area. They were the share of deaths by suicide and the rate of individuals suffering from chronic mental disorders assessed by individuals covered by the long-term illness scheme for psychiatric reasons per 100,000 inhabitants, which provides an estimation of the treated prevalence of chronic disorders. We also included characteristics of the overall health status of the population. Somatic comorbidities can indeed lead to increased mental health care needs due to additive and interactive effects of comorbid physical and mental conditions [43] and individuals suffering from multiple chronic conditions use health care more frequently than other patients [44]. Furthermore, individuals with mental illnesses represent a high share of individuals with chronic somatic conditions [45]. We therefore considered the global mortality rate, the rate of individuals covered by the long-term illness scheme for somatic disorders and the acute admission rate for somatic disorders. These variables were extracted from the database of the national centre on epidemiological causes of death (*Centre d’épidémiologie sur les causes médicales de décès*), the census database (*Base des recensements de la population*), the French national discharge database for somatic care (*Programme de médicalisation des systèmes d’information en médecine, chirurgie, obstétrique*) and the Eco-Santé database which provides data on the health of the French population before 2016 [46–49].

Second, we considered the demographics of the population, which have been shown to be correlated with health needs [19]. We included the percentage of women in the adult population of sectors’ catchment areas and the mean age of this population, extracted from the census database [48].

Finally, socio-economic factors have also been shown to be correlated with health needs [50, 51]. In order to account for such characteristics, we calculated a proxy deprivation index for each zip code belonging to a sector’s catchment area and calculated its mean value on the area. This index, named FDep, was specifically developed for the French context and takes into account the median household income, the percentage of high school graduates in the population aged 15 years and older, the percentage of blue-collar workers (individuals who perform manual labour) in the active population and the unemployment rate [52–54].

#### Factors related to the supply of health and social care

Factors potentially associated with variations in inpatient admission rates in sectors’ catchment areas that were of particular interest in our study were factors related to the supply of health and social care as they can be targeted by dedicated policies to reduce unwarranted geographic variations in mental health care [55]. We first considered characteristics of the supply of public mental health care. We included both institutional characteristics (public or private non-profit status, specialization or not in psychiatry and participation or not to teaching activities and to emergency care) and organizational characteristics (number of psychiatric inpatient beds) of the hospital to which each psychiatric sector was linked.

Second, we considered characteristics of the supply of private mental health care in sectors’ catchment area. We included the availability of self-employed community-based psychiatrists or psychologists and of hospitalization beds in private psychiatry.

Third, we considered the availability of non-specialized health care, both for primary care (general practitioners) and for hospital-based care (non-psychiatric hospitalization beds).

Finally, we included data on the supply of social care (number of beds in housing institutions for disabled individuals, capacity of centres providing care through employment and capacity of housing and social rehabilitation centres).

The full list of supply factors considered is available in Table 1. Information regarding these variables was extracted from complementary national administrative databases: the SAE database, the French national database of permanent facilities (*Base permanente des équipements*), the national register of health and social institutions (*Fichier national des établissements sanitaires et sociaux*) and the national directory of professionals [35, 56–58]. The density of the different supply factors was then calculated for the catchment area of each sector.

**Table 1:** Characteristics of care supply in sectors’ catchment areas

Supply characteristic	N (%) or mean (SD)
Supply of public mental health care (*characteristics of the hospital to which each sector was linked*)	
Public hospitals	170 (96.59)
Hospitals specialized in psychiatry	73 (41.48)
Hospitals participating to teaching activities	18 (10.23)
Hospitals participating to emergency care	159 (90.34)
Number of inpatient beds in the hospital (per 100,000 inhabitants)	29.43 (17.34)
Supply of private mental health care (*per 100,000 inhabitants*)	Mean (SD)
Number of community-based private psychiatrists	12.75 (9.64)
Number of psychologists	70.77 (46.05)
Number of psychiatric inpatient beds in private for-profit hospitals	27.74 (22.57)
Supply of non-specialized health care (*per 100,000 inhabitants*)	Mean (SD)
Number of general practitioners	108.22 (20.24)
Number of non-psychiatric inpatient beds	2170.68 (390.46)
Supply of social care (*per 100,000 inhabitants*)	Mean (SD)
Number of beds in housing institutions for disabled individuals	195.43 (80.46)
Capacity of centres providing care through employment	203.20 (63.72)
Capacity of housing and social rehabilitation centres	100.64 (41.29)

#### Level of urbanization

Urbanicity is likely to be associated with psychiatric care through different mechanisms related to coordination and travel distances to the different types of care supply [59]. We therefore also considered the level of urbanization as a factor potentially associated with geographic variations in inpatient admission rates. This characteristic was assessed by the density of inhabitants in the zip codes of the sectors’ catchment areas and extracted from a national administrative database on urbanicity (*Base des unités urbaines*) [60].

### Analysis

#### Description of variations

After summarizing the characteristics of sectors’ catchment areas (both in terms of population characteristics and availability of care supply), either by the mean and standard deviation (SD) or by number (%), we described variations in inpatient admission rates per 100,000 inhabitants between psychiatric sectors’ catchment areas. We calculated the national mean, SD, median, interquartile range and range. A coefficient of variation (CV), which measures the dispersion around the national mean [61], was interpreted together with the ratio between the 90th and the 10th percentiles of the distribution of each variable, which is less sensitive to outlier values [55, 62]. To identify the potential impact of these outlier values, we built a boxplot of the inpatient admission rate as well as a waterfall plot representing this rate in each sector’s catchment area, ranked by decreasing order, in comparison to the national average.

#### Identification of factors associated with variations in inpatient admission rates

To identify characteristics of the supply of health and social care in sectors’ catchment areas which were associated with variations in inpatient admission rates after adjustment for other relevant factors, we modelled the observed number of inhabitants in each sector’s catchment area admitted at least once in inpatient care over the course of the year 2012 (event) through a negative binomial regression. This was preferred to a conventional Poisson model due to overdispersion in the count data (deviances considerably exceeding the degrees of freedom). The natural logarithm of the total number of inhabitants aged over 16 in each sector’s catchment area (expected number of events) was used as an offset term in the regression [23, 63, 64]. All supply factors presented above were introduced as explanatory variables. We also added factors relating to epidemiological data, as adjustment factors, as well as the level of urbanization. When explanatory variables were highly correlated or associated, only one of them was kept in the model based on the strength of association with the inpatient admission rate and clinicians’ advice. The analysis produced estimated values of the regression coefficients for all explanatory variable in the model, whose sign indicated whether the association was positive or negative, as well as their 95% confidence intervals (95% CI). Statistical significance was taken to be indicated by a probability value of 0.05 or less. Regression coefficients for each explanatory variable were finally exponentiated to obtain an estimation of the inpatient admission incidence rate ratio given the other variables were held constant in the model. The rate ratio for the dependent variable was expressed for a one unit increase in each continuous explanatory variable while, for categorical explanatory variables, a reference was chosen for comparison [63, 64].

All the analyses were performed using SAS software version 9.4 (SAS Institute Inc., Cary, NC, USA).

## Results

### Setting

The data of 531 adult psychiatric sectors, accounting for 66.1% of all adult psychiatric sectors reported in the RIM-P database of the year 2012 for mainland France, were included in the analysis based on the availability of exhaustive inpatient admissions data in the hospital to which they were linked. Such hospitals (*n* = 176) represented 71.0% of all hospitals participating in psychiatric sectorisation registered in the RIM-P database. Hospitals whose data was included and hospitals whose data was excluded did not differ significantly in terms of main institutional, organisational or case-mix characteristics (see Additional file 1), with the exception of the percentage of patients diagnosed as suffering from psychotic disorders other than schizophrenia which was lower in hospitals whose data was included (7.5% vs. 8.7%, *p* = 0.031).

### Characteristics of sectors’ catchment areas

#### Characteristics of the population

The mean percentage of women of sectors’ catchment areas was 52.3% (±2.7) and the mean age of this population was 48.1 years old (±3.8). On average, the number of individuals suffering from chronic mental disorders per 100,000 inhabitants of sectors’ catchment areas was 1627.1 (±366.9) and deaths by suicide represented 5.1% (± 2.1%) of all deaths.

Six hundred and twenty four thousand, nine hundred and seventeen individuals living in these catchment areas were treated in the corresponding psychiatric sectors in 2012 (corresponding to 5,573,778 admissions to the different types of mental health care). Among them, 140,578 individuals were admitted at least once in inpatient care. The majority (54.2%) had only one inpatient admission over the course of the year 2012 and the median number of inpatient admissions per patient was equal to 1.0.

#### Characteristics of care supply

Overall, sectors whose catchment area was included in the analysis were mostly linked to public general hospitals. Characteristics of care supply available in these catchment areas are described in Table 1.

### Variations in inpatient admission rates

Considerable variations in the inpatient admission rate per 100,000 inhabitants were observed between sectors’ catchment areas with rates ranging from 1.1 to 498.3 per 100,000 inhabitants and a coefficient of variation reaching 77.7%. The value of the ratio between the 90th and the 10th percentiles of the distribution was also high suggesting that variations remained considerable even when not taking into account outlier values (Table 2).

**Table 2:** Variations in inpatient admission rate between psychiatric sectors’ catchment areas

	Mean (SD)	Median (interquartile range)	Range	CV (%)	Ratio 90/10th percentiles
Inpatient admission rate per 100,000 inhabitants	77.26 (60.04)	63.32 (66.49)	497.20	77.71	7.65

**Fig. 1:**
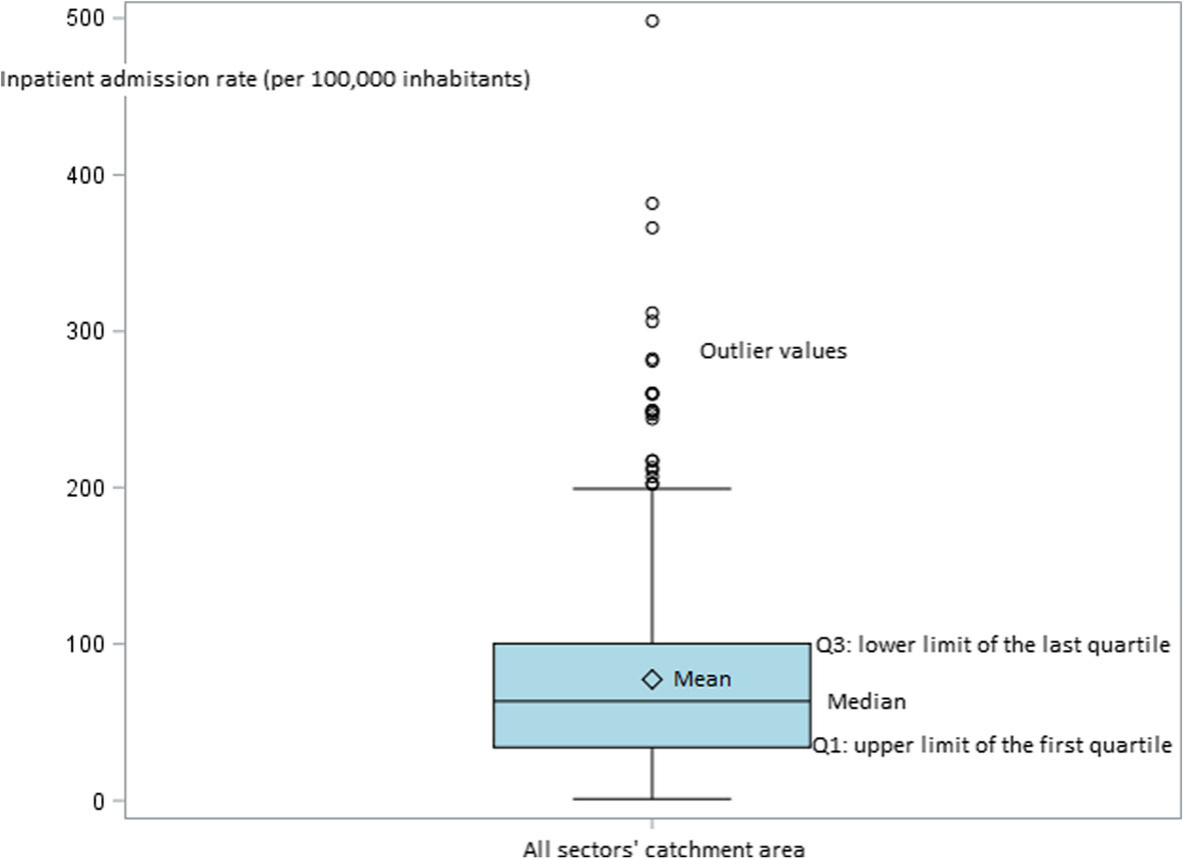
Boxplot of the inpatient admission rate in sectors’ catchment areas

**Fig. 2:**
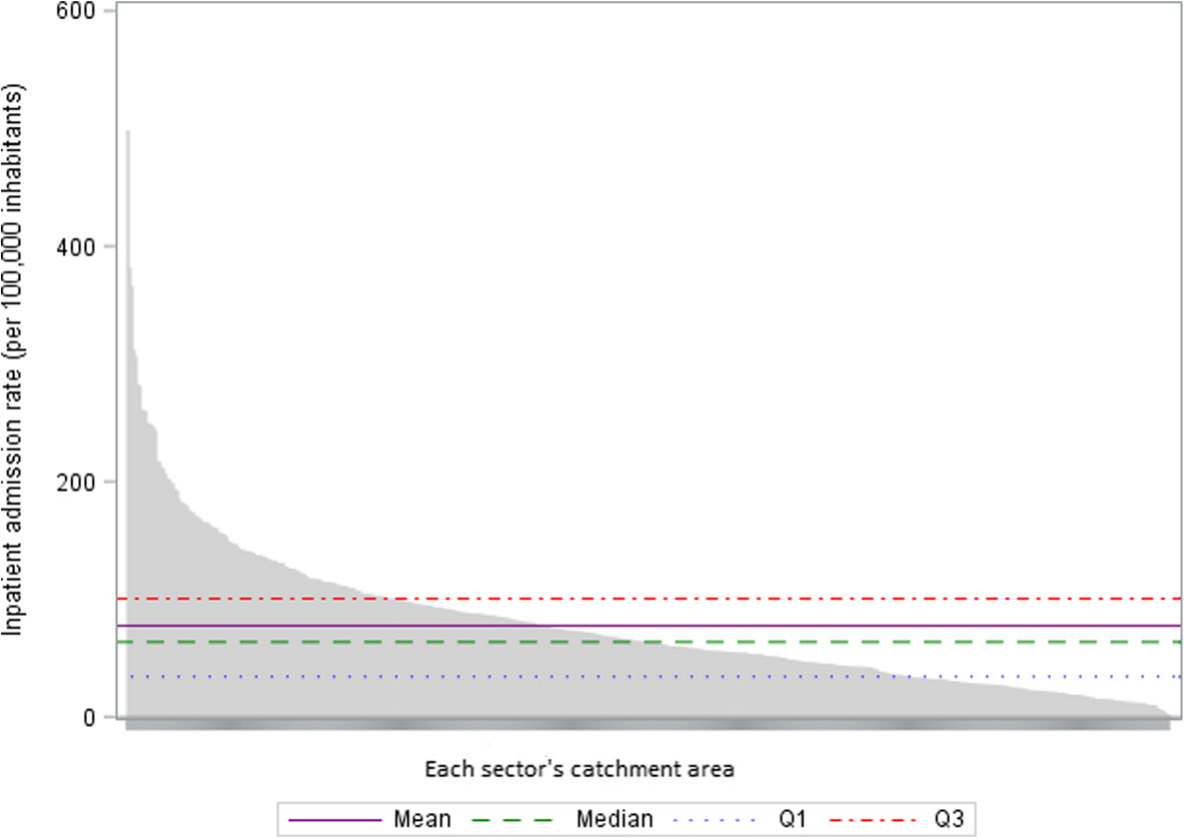
Inpatient admission rate in each psychiatric sector’s catchment area in comparison to the national average. Q1: upper limit of the first quartile; Q3: lower limit of the last quartile NB: Each point on the x-axis corresponds to a different sector’s catchment area

This was confirmed by the graphical approach as a limited number of outlier values appeared on the boxplot of the inpatient admission rate in sectors’ catchment areas (Fig. 1) while the waterfall plot showed a wide scattering of the values of the inpatient admission rate (not only for a limited number of catchment areas) in comparison to aggregated national values (Fig. 2).

### Factors associated with variations in inpatient admission rates

After adjusting for epidemiological differences, several characteristics of the supply of care in sectors’ catchment areas were significantly associated with the inpatient admission rate per 100,000 inhabitants in the negative binomial regression.

Regarding the characteristics of public mental health care, the fact that a sector was linked to a private non-profit hospital (vs. a public hospital) and to a hospital participating to emergency care was associated with an increase in the inpatient admission rate of respectively 63.9 and 24.8%. On the contrary, the fact that a sector was linked to a hospital participating to teaching activities and to a hospital specialised in psychiatry (vs. a general hospital) was associated with a significant decrease in the inpatient admission rate of respectively 32.5 and 29.4% (Table 3).

**Table 3:** Results of the multivariable analysis (negative binomial regression)

Variable	Estimated value of the coefficient	Standard error	95% Confidence interval of the coefficient		Exponentiated coefficient	*P*-value
			Lower bound	Upper bound		
Intercept	−9.6329	0.9758	−11.5455	−7.7203		**< 0.0001**
Epidemiological characteristics						
Psychiatric health status of the population						
Number of individuals suffering from chronic mental disorders (per 100,000 inhabitants)^a^	0.0000	0.0001	−0.0002	0.0003	1.0000	0.8069
Percentage of deaths by suicide among total deaths	0.0837	0.0283	0.0282	0.1391	1.0873	**0.0031**
Overall health status of the population (per 100,000 inhabitants)						
Acute admission rate for somatic disorders	0.0000	0.0000	−0.0000	0.0000	1.0000	0.2079
Mortality rate	0.0014	0.0009	−0.0004	0.0032	1.0014	0.1223
Number of individuals suffering from chronic somaticdisorders	0.0000	0.0000	−0.0001	0.0000	1.0000	0.1868
Demographics of the population						
Number of women (per 100,000 inhabitants)	0.0000	0.0000	−0.0000	0.0000	1.0000	0.9899
Mean age of individuals aged over 16	0.0166	0.0163	−0.0155	0.0486	1.0167	0.3109
Socio-economic characteristics of the population						
Quintile of the mean deprivation index (FDep) (from lower to higher deprivation), reference: 5th quintile						
1	0.2679	0.1399	−0.0064	0.5422	1.3072	0.0556
2	0.2941	0.1117	0.0751	0.5130	1.3419	**0.0085**
3	0.2939	0.1102	0.0779	0.5098	1.3416	**0.0076**
4	0.2944	0.1050	0.0886	0.5002	1.3423	**0.0051**
Characteristics of the supply of health and social care						
Supply of public mental health care						
*Characteristics of the hospital to which each sector was linked*						
Private non-profit (vs. public)	0.4939	0.1990	0.1039	0.8840	1.6387	**0.0131**
Participation to teaching activities (vs. no participation)	−0.3929	0.1001	−0.5891	−0.1968	0.6751	**< 0.0001**
Specialization in psychiatry (vs. general hospital)	−0.3476	0.0721	−0.4890	−0.2062	0.7064	**< 0.0001**
Participation to emergency care (vs. no participation)	0.2215	0.1068	0.0122	0.4307	1.2479	**0.0380**
Number of inpatient beds in the hospital (per 100,000 inhabitants)^c^	0.0046	0.0025	−0.0002	0.0095	1.0046	0.0609
Supply of private mental health care (per 100,000 inhabitants)						
Number of community-based private psychiatrists^b^	−0.0202	0.0055	−0.0310	−0.0094	0.9800	**0.0003**
Number of psychologists	0.0011	0.0008	−0.0005	0.0026	1.0011	0.1678
Number of psychiatric inpatient beds in private for-profit hospitals^a^	−0.0013	0.0020	−0.0052	0.0026	0.9987	0.5059
Supply of non-specialized care (per 100,000 inhabitants)						
Number of general practitioners^b^	−0.0009	0.0023	−0.0054	0.0035	0.9991	0.6777
Number of non-psychiatric inpatient beds	0.0001	0.0001	−0.0001	0.0002	1.0001	0.5699
Supply of social care (per 100,000 inhabitants)						
Number of beds in housing institutions for disabled individuals	0.0012	0.0006	0.0001	0.0023	1.0012	**0.0335**
Capacity of centres providing care through employment	0.0003	0.0007	−0.0010	0.0017	1.0003	0.6101
Capacity of housing and social rehabilitation centres	−0.0001	0.0008	−0.0017	0.0015	0.9999	0.8884
Level of urbanization						
Level of urbanization (from lower to higher urbanization), reference: 6th quantile						
1	−0.0818	0.1147	−0.3066	0.1429	0.9215	0.4754
2	−0.0664	0.1448	−0.3503	0.2174	0.9358	0.6463
3	−0.0456	0.3700	−0.7709	0.6796	0.9554	0.9018
4	−0.1111	0.5310	−1.1518	0.9296	0.8948	0.8343
5	−0.1557	0.1118	−0.3749	0.0634	0.8558	0.1637

^a^ and ^b^ Significant correlations were observed between these variables. However, corresponding correlation coefficients were weak and there were strong hypotheses on associations of these variables with psychiatric inpatient admission rates so they were all introduced in the model. ^c^The number of inpatient beds per 100,000 inhabitants of the catchment area was highly correlated with the total number of full-time equivalents allocated to psychiatric care by the hospital to which each sector was linked per 100,000 inhabitants of the catchment area. We therefore only introduced the number of beds in the model

Considering the characteristics of private mental health care, an increase by 1.0 in the number of community-based private psychiatrists per 100,000 inhabitants, was associated with a 2.0% decrease in the inpatient admission rate (Table 3).

No association was found with any of the characteristics of non-specialised care (Table 3).

Finally, regarding the availability of social care on sectors’ catchment areas, an increase by 1.0 in the number of beds in housing institutions for disabled individuals per 100,000 inhabitants was associated with an increase by 0.1% of the inpatient admission rate (Table 3).

Notably, considering our adjustment factors, while a higher share of deaths by suicide on the catchment area was significantly associated with an increase in the inpatient admission rate, this was not the case of the rate of individuals suffering from chronic mental disorders (Table 3).

## Discussion

Considerable variations in inpatient admission rates were observed between sectors’ catchment areas and were widespread on the French territory. Characteristics of the supply of care were associated with these variations, even after adjusting for epidemiological differences and varying levels of urbanization. Notably, significant associations were found with the institutional characteristics of the hospital to which each sector was linked. Similarly, an increase in the availability of community-based private psychiatrists was associated with a decrease in the inpatient admission rate while an increase in the capacity of housing institutions for disabled individuals was associated with an increase in this rate.

If recent data regarding variations in inpatient admission rates in psychiatry are scarce, considerable variations in those rates have also been underscored in other countries [19–22]. In studies where it was available, the ratio between the highest and the lowest rates was inferior to five. This is lower than what we found in our findings. However, other works have considered rates standardised on the age and sex structure of the population in the descriptive steps [21, 22]. At the French national level, the variations observed for the psychiatric inpatient admission rate can be compared to those of the inpatient admission rate in other medical specialties. They were systematically more significant (in terms of both coefficient of variation and ratio between the 90th and the 10th percentiles of the distribution) than those observed for a wide range of specific somatic medical procedures [65]. These differences could be linked to the particularities of psychiatry where clinical uncertainty remains [66–68]. In addition, geographic areas used to study variations were smaller in our study and variations could tend to homogenise on larger areas.

The width of the variations in inpatient admission rates observed between psychiatric sectors’ catchment areas question appropriateness of care and suggest that some of them may be unwarranted. This hypothesis is further supported by the significant associations found between these variations and the characteristics of the supply of care in sectors’ catchment area, even after adjusting for epidemiological differences. Several hypotheses can be made on the underlying mechanisms which could explain these associations. The associations found between the institutional characteristics of the hospital to which was linked each sector and the inpatient admission rate may be explained by varying practice patterns between the different types of hospitals which should be further explored. The negative association found between the availability of community-based private psychiatrists and the inpatient admission rate may be explained by a better continuity of mental health care when alternatives in the community are available which may reduce the need for inpatient admission. The positive association found between the number of beds in housing institutions for disabled individuals and the psychiatric inpatient admission rate may be linked to the fact that individuals with chronic, severe and disabling mental disorders tend to live in areas where the supply of social care is high [19].

Several policy and organizational implications can be drawn from our findings. They underscored that geographic variations in psychiatric inpatient admission rates are a reality that merits the full attention of decision makers and should be monitored in routine. In addition, they were associated with characteristics of the supply of care which represent a lever on which policy makers can act quickly by designing and implementing dedicated measures. Our findings advocate for a homogenous repartition of health and social care for mental disorders in sectors’ catchment areas in lines with the health needs of the population served. This should apply particularly to community-based private psychiatrists whose availability was associated with variations in the use of inpatient care in our study and whose heterogeneity of repartition on the French territory has often been underscored [69].

The strengths of our study, which focused on an under-explored area of research, rely on its national scale and the inclusion of a wide range of characteristics potentially associated with variations in psychiatric inpatient admission rates. In particular, we considered supply factors relating to both public and private care, health and social care, hospital and community-based care, and specialised and non-specialised care. However, our results should be interpreted in consideration of several limitations. First, we had to exclude the data of some sectors linked to hospitals for which exhaustive information on their inpatient admissions were not available in order not to interpret the variability in data reporting and quality as variability in inpatient admission rates. However, such hospitals presented very few differences with hospitals whose data was included. Second, we were limited by the lack of detailed organisational characteristics of psychiatric sectors in the databases used. Third, we did not have access to detailed information related to the prevalence of the different mental disorders in sectors’ catchment areas as such information is currently not available. We therefore had to use a proxy, the rate of individuals covered by the long-term illness scheme for psychiatric reasons, which is limited to people who are already in contact with the healthcare system. Finally, our findings could be usefully complemented by additional research focusing on the frequency and intensity of inpatient admissions, in particular by accounting for multiple admissions.

## Conclusions

Our results demonstrate significant variations in inpatient admission rates between psychiatric sectors’ catchment areas at the national level in France. These variations were associated with characteristics of the supply of care which suggest that a policy leeway remains to reduce these variations in parallel to the development of interventional research.

## Additional file

Additional file 1: Main characteristics of hospitals whose data was included or excluded. (DOCX 16 kb)

## Abbreviations

95% CI: 95% confidence interval
CNIL: French data protection authority
CV: Coefficient of variation
FDep: French deprivation index
ICD-10: International Classification of Diseases, tenth revision
Q1: Upper limit of the first quartile
Q3: Lower limit of the last quartile
RIM-P: *Recueil d’informations médicalisé en psychiatrie*, French national psychiatric discharge database
SAE: *Statistique annuelle des établissements de santé*, French annual national survey on health care providers
SD: Standard deviation
SHI: Social health insurance
