# Supplementary material for: Understanding geographic variations in psychiatric inpatient admission rates: width of the variations and associations with the supply of health and social care in France
Source: BMC Psychiatry. 2018 Jun 5;18:174. doi: 10.1186/s12888-018-1747-2 (PMC5989448; doi:10.1186/s12888-018-1747-2)
Supplement: Supplementary file 1 — Main characteristics of hospitals whose data was included or excluded. (DOCX 16 kb) [file 12888_2018_1747_MOESM1_ESM.docx]

**Additional file 1. Main characteristics of hospitals whose data was included or excluded**

| **Characteristic** | **Hospitals whose data was included** (n=176) | **Hospitals whose data was excluded** (n=72) | **P-value** (difference between included and excluded hospitals) |
| --- | --- | --- | --- |
| ***Characteristics of the case-mix*** | | | |
| *Demographic characteristics* | | | |
| Mean age | 47.6 | 46.7 | 0.1568 |
| % of female patients | 54.4 | 53.8 | 0.1825 |
| *Clinical characteristics** | | | |
| % of patients with a diagnosis of addiction (ICD-10 codes: F10-F19) | 12.7 | 11.9 | 0.5966 |
| % of patients with a diagnosis of schizophrenia (ICD-10 code: F20) | 14.7 | 15.4 | 0.1264 |
| % of patients with a diagnosis of psychotic disorders (ICD-10 codes: F21-F29) | 7.5 | 8.7 | **0.0310** |
| % of patients with a diagnosis of bipolar disorders (ICD-10 code : F31) | 27.4 | 24.2 | 0.0785 |
| % of patients with a diagnosis of mood disorders other than bipolar disorders (ICD-10 codes: F30, F32, F33, F34, F38, F39) | 7.4 | 7.8 | 0.4682 |
| % of patients with anxiety disorders (ICD-10 codes: F40-F48) | 29.0 | 26.9 | 0.1405 |
| % of patients with other mental or behavioural disorders (ICD-10 codes: F50-F59, F60-F69, F84, F90-F98, F99) | 17.5 | 16.7 | 0.2090 |
| ***Institutional characteristics*** | | | |
| % of public hospitals | 96.6 | 90.3 | 0.4290 |
| % of hospitals specialized in psychiatry | 41.5 | 36.1 | 0.4345 |
| % of hospitals participating to teaching activities | 10.23 | 12.50 | 0.6020 |
| ***Organisational characteristics*** | | | |
| Number of inpatient psychiatric beds | 150.7 | 174.2 | 0.2784 |

***** Patients could have more than one diagnosis over the course of the year 2012
